# Minutia Tensor Matrix: A New Strategy for Fingerprint Matching

**DOI:** 10.1371/journal.pone.0118910

**Published:** 2015-03-30

**Authors:** Xiang Fu, Jufu Feng

**Affiliations:** Key Laboratory of Machine Perception (MOE), Department of Machine Intelligence, School of Electronics Engineering and Computer Science, Peking University, Beijing, China; Beihang University, CHINA

## Abstract

Establishing correspondences between two minutia sets is a fundamental issue in fingerprint recognition. This paper proposes a new tensor matching strategy. First, the concept of minutia tensor matrix (simplified as *MTM*) is proposed. It describes the first-order features and second-order features of a matching pair. In the *MTM*, the diagonal elements indicate similarities of minutia pairs and non-diagonal elements indicate pairwise compatibilities between minutia pairs. Correct minutia pairs are likely to establish both large similarities and large compatibilities, so they form a dense sub-block. Minutia matching is then formulated as recovering the dense sub-block in the *MTM*. This is a new tensor matching strategy for fingerprint recognition. Second, as fingerprint images show both local rigidity and global nonlinearity, we design two different kinds of *MTM*s: local *MTM* and global *MTM*. Meanwhile, a two-level matching algorithm is proposed. For local matching level, the local *MTM* is constructed and a novel local similarity calculation strategy is proposed. It makes full use of local rigidity in fingerprints. For global matching level, the global *MTM* is constructed to calculate similarities of entire minutia sets. It makes full use of global compatibility in fingerprints. Proposed method has stronger description ability and better robustness to noise and nonlinearity. Experiments conducted on Fingerprint Verification Competition databases (*FVC2002* and *FVC2004*) demonstrate the effectiveness and the efficiency.

## Introduction

Fingerprint matching is a classical and hot topic in computer vision and pattern recognition [1]. Researchers have set up a series of special fingerprint verification competition databases [2] [3]. Establishing correspondences between fingerprint minutia sets is a fundamental issue in fingerprint recognition. It is challenging to find perfect correspondences for fingerprints due to various reasons: non-linear distortion, partial overlap, noise and so on.

Many minutia-based fingerprint matching papers have been published these years. They can be mainly classified into two categories. The first category of these papers is to exact more matching features besides minutia locations and orientations. Jain et al. used pores and ridge contours besides minutia points and proposed a three-level (level 1: pattern, level 2 minutia points and level 3: pores and ridge contours) matching strategy [4]. Liu et al. proposed the minutiae phase difference feature to describe the minutiae [5]. Choi et al. incorporated ridge features with minutiae and achieved good results [6]. Izadi et al. introduced cylinder quality measure into minutia cylinder-code based fingerprint matching [7]. Wen et al. applied a set of global level minutia dependent features, including minutia qualities and the area of overlapping regions [8]. Thuy et al. increased the ridge-valley structure features and improved the classical Thin-Plate-Spline deformation model [9], and they proposed the local Thin-Plate-Spline to deal with non-linear distorted fingerprints [10]. In summary, these papers are trying to seek for more matching features to improve the effectiveness.

The other category of these papers just uses the minutia features and tries to construct more complex structures or to find more efficient algorithms. Since only the minutia features are used, this category of approaches faces greater challenges. Previous pioneers have achieved big progress. Ratha et al. proposed a local similarity calculation algorithm for local matching [11]. Chen et al. constructed the minutia topological structure to increase the tolerance for noise [12]. Chikkerur et al. proposed K-PLET structures and a graph-based representation for minutia matching [13]. Xu et al. grew minutia triangle structures into a growing area and then performed the global match by fusing them[14]. Cappelli et al. proposed a novel representation based on 3D cylinder structures, called Minutia Cylinder-Code [15]. Medina-Pérez et al. improved fingerprint verification by using minutia triplets [16]. In summary, minutia topologic structures constructed in local areas are considered to be less impacted by nonlinear distortion and noise. However, as the radius of minutia structures is not big, it limits the use of global minutia information. Based on this consideration, some researchers try to introduce global information into the matching process. Tea et al. presented an approach based on convex hull to deal with incomplete or partial fingerprints [17]. Feng et al. proposed the compatibility of local minutia pairs and adopted a relaxation iterative process [18]. Cao et al. applied compatibility to local minutia star-structures and achieved better results [19]. All these papers are trying to construct more complex minutia topologic structures or seek for more efficient matching strategies.

Establishing correspondences between two feature sets is a fundamental issue in computer vision and pattern recognition [20]. Horaud et al. proposed a graph matching framework and formulated point matching as recovering the maximal cliques in the correspondence graph [21]. Duchenne et al. proposed the affinity tensors to describe high-order affinities between each feature pairs [22]. First-order tensor describes similarities of each feature pair. Second-order tensor describes compatibilities between each two feature pairs. Leordeanu et al. proposed a spectral technique for correspondence problems using pairwise constraints [23]. They recovered the matching relationship based on spectral matching methods, by using the principal eigenvector of adjacency matrix and imposing the one-to-one mapping constraints. This notion can be introduced into minutia matching.

Based on these insights, we propose a tensor matching strategy. We construct the minutia tensor matrix (simplified as *MTM*) for fingerprint minutiae. It unifies both the first-order features and the second-order features. The diagonal elements in *MTM* indicate similarities of each minutia pair and other elements indicate pairwise compatibilities between minutia pairs. Correct minutia pairs are more likely to establish both large similarities within them and large compatibilities among them, thus they form a dense sub-block. Incorrect pairs establish links with the other pairs accidentally, so they are unlikely to belong to dense sub-blocks (The definition of dense sub-block can be seen in section 2.1). Minutia matching is formulated as recovering the main dense sub-block in the *MTM*.

Our previous papers tried to apply the tensor idea to fingerprint matching [24] [25]. We applied the spectral matching strategy to fingerprint global matching [24]. The disadvantage was that it was only applied to global matching, while other strategies were still required for local matching. We proposed extended clique models to deal with local matching and global matching [25]. The disadvantage is that the local matching process is time-consuming. Meanwhile, local matching is not associated with global matching. This paper is innovative on the basis of previous papers. First, the tensor strategy is used in local matching for the first time. Second, by use of the proposed “*MTM*”, local matching and global matching are unified into an integrated framework.

Main contributions of this paper are: First, **the proposed *MTM* unifies both similarities and compatibilities appropriately**. Second, minutia matching is formulated as recovering the dense sub-block in the *MTM*. Optimal matching relationship corresponds to the dense sub-matrix in the *MTM*. **It gives a clear mathematical meaning for “optimal matched pairs”**. Third, **spectral matching methods are then used for recovering the dense block. It is efficient and effective**, which can be seen in the following experiments. Forth, **two *MTM*s with different constraints are constructed, which makes use of local rigidity and global compatibility, respectively**. In the local matching level, we build the local minutia topologic structures and construct local *MTM* for each minutia structure pair. Calculating similarities of minutia pairs corresponds to recovering the dense sub-block in the local *MTM*. In the global matching level, we construct the global *MTM* for entire minutia sets. Calculating similarities of minutia sets corresponds to recovering the dense sub-block in the global *MTM*. Proposed approach has stronger description ability and better robustness to non-linear deformation and noise.

## Materials and Methods

### 2.1 Problem formulation

Suppose there are two fingerprint minutia sets *P* and *P'*, with *N*_*p*_ and *N*_*p'*_ minutiae, respectively. We define two attributed graphs *G*_*P*_ = (*V*,*E*,*A*) for minutia set *P* and *G*_*P'*_ = (*V'*,*E'*,*A'*) for minutia set *P'*. Each edge e = ij ∊ E in *G*_*P*_ is assigned an attribute *A*_*ij*_, which is the distance vector between minutia *i* and minutia *j* in *P*. We represent node attributes as special edge attributes, i.e. *A*_*ii*_ for node *i*. So it is with *P'*. Let *S*_*A*_(*ii'*, *jj'*) indicate the similarity score between attributes *A*_*ij*_ and *A'*_*i'j'*_. We want to find a mapping that best preserves the attributes of nodes and edges between attributed graphs *G*_*P*_ and *G*_*P'*_. Equivalently, we seek for a set of correct minutia pairs *M* = (*ii'*,*jj'*,*kk'*…) so as to maximize the matching score, defined as:

M~=argmaxM∑ii',jj'∈MSA(ii',jj') (1)

High-order tensors are constructed to represent high-order affinities [22]. Inspired by this notion, we propose the minutia tensor matrix (*MTM*). It is proposed based on these considerations: First-order tensor matrix describes similarities of each minutia pair. Second-order tensor matrix describes compatibilities between each two minutia pairs. For fingerprint minutia sets *P* and *P'*, each minutia pair (*i*, *i'*) is assigned an similarity attribute *T*_1_ (*i*, *i'*) and each two minutia pairs (*ii'*, *jj'*) is assigned a compatibility attribute *T*_2_ (*ii'*, *jj'*). (Calculation of *T*_1_ (*i*, *i'*) and *T*_2_ (*ii'*, *jj'*) can be seen in section 2.3 and 2.4). Thus we can build the first-order tensor matrix *T*_1_ and the second-order tensor matrix *T*_2_. Here we don’t use tensor matrixes with three or more orders as the computation is unacceptable. After that we fuse *T*_1_ and *T*_2_ into a fused high-order tensor matrix *T*_F_, which is also the proposed *MTM*. The fusion rule is shown in the formula:

$$T_{F}\left(ii^{\prime },jj^{\prime }\right)=\{\begin{array}{cc} 0 & if\;\left(i=j\;and\;i^{\prime }\neq j^{\prime }\right)\;or\;\left(i\neq j\;and\;i^{\prime }=j^{\prime }\right)\\ T_{1}\left(i,i^{\prime }\right) & \;if\;i=i^{\prime }\;and\;j=j^{\prime }\\ \lambda \cdot T_{2}\left(ii^{\prime },jj^{\prime }\right) & \;\;else \end{array}$$ (2)

Where *T*_1_ is the first-order tensor matrix, whose element *T*_1_ (*i*, *i'*) describes the similarity of minutia pair (*i*, *i'*). *T*_2_ is the second-order tensor matrix, whose element *T*_2_ (*ii'*, *jj'*) describes compatibility between (*i*, *i'*) and (j, *j'*). The fused tensor matrix *T*_*F*_ (*MTM*) is a two-dimensional matrix with (*N*_*p*_ *× N*_*p'*_) × (*N*_*p*_ *× N*_p'_) elements. It unifies both similarities and compatibilities among minutia pairs. When the condition (*i = i' and j = j'*) holds, minutia pairs (*i*, *i'*) and (j, *j'*) are the same pair, thus *T*_1_ (*i*, *i'*) = *T*_1_ (*j*, *j'*). As minutia matching is one-to-one matching, minutia *i* in *P* mapped to both minutia *i'* and *j'* in *P'* is impossible. When (*i = j and i' ≠ j'*) or (*i ≠ j and i' = j'*) holds, *T*_2_ (*ii'*, *jj'*) is 0.We use this priori knowledge to prune and can get a very sparse *MTM*. The parameter λ is a weighting factor. As *T*_1_ and *T*_2_ have different dimensions, we use *λ* to adjust the values. It is set manually. *T*_*F*_ is a symmetric matrix as *T*_*F*_ (*ii'*, *jj'*).

**Fig 1:**
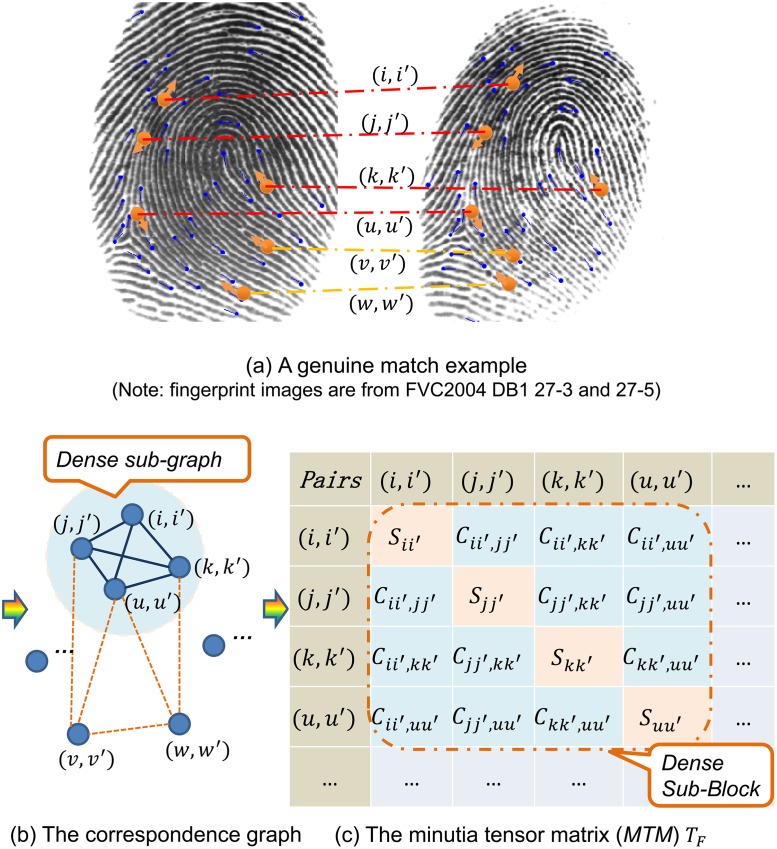
Illustration of the main idea in our method. The fingerprint images are from FVC2004 DB1 27–3 and 27–5. For clarity, only a small subset of minutia pairs are shown. Candidate minutia pairs shown in (a) form the correspondence graph in (b) and the minutia tense matrix (*MTM*) in (c). Genuine minutia pairs corresponds to the dense subgraph of correspondence graph, and also the dense block of *MTM*. Minutia matching is formulated as recovering the dense sub-block in the *MTM*. It can be solved by the spectral correspondence methods.

As shown in Fig. 1, for two minutia sets *P* and *P'*, we can build the correspondence graph *G*_*PP'*_. In graph *G*_*PP'*_, the dense subgraph *G*_*A*_ is the biggest strong-connected subgraph in *G*_*PP'*_, whose nodes have strong links with all the other nodes. Correct minutia pairs are likely to establish links among each other and thus form a strongly connected subgraph, which is also the dense subgraph. Incorrect pairs establish links with the other pairs only accidentally. Minutia matching can be formulated as seeking for the dense subgraph in the correspondence graph.

The correspondence graph *G*_*PP'*_ corresponds to the *MTM T*_*F*_. In *T*_*F*_, the dense sub-block *T*_*D*_ is the biggest sub-matrix in *T*_*F*_, whose elements in all the rows and all the columns have big values. The element values in the dense sub-block *T*_*D*_ should be greater than a certain threshold. As shown in Fig. 1, for minutia sets *P* and *P'*, we can build the *MTM T*_*F*_ of the correspondence graph *G*_*PP'*_. Correct pairs are likely to establish elements with big values among each other and thus form a strongly connected cluster, which is also the dense sub-block *T*_*D*_. Minutia matching corresponds to recovering the dense sub-block.

Equivalently, we seek for a set of correct matching pairs *M* = (*ii'*, *jj'*, *kk'*…), so as to maximize the *MTM* score, defined as:

M~=argmaxM∑ii'∈M,jj'∈MTF(ii',jj') (3)

The main idea is illustrated in Fig. 1. The optimization problem (3) can be solved by spectral matching method, which will be discussed in section 2.2.

### 2.2 Spectral matching methods

Formula (3) yields to the following binary optimization problem:

m~=argmaxm(mTTFm),m∈{0,1}pq,s.t. m1≤1 and mT1≤1 (4)

The optimal solution $\tilde{m}$ is the binary vector that maximizes the *m* ^*T*^ *T*_*F*_ *m* score. *m* is a row-wise vectored replica of the *MTM T*_*F*_. It can be derived by using the principal eigenvector of *T*_*F*_ and imposing the one-to-one mapping constraints, which is proposed in [23].

Here we make some improvements. Firstly we prune the elements with small similarities or low compatibilities. Secondly we relax both the mapping constraints and the integral constraints on *m*, so that its elements can take real values in [1]. Thirdly we don’t initialize *m*_0_ (the initialization value of *m*) randomly like [23], we use some priori knowledge. As minutia pairs with big local structural similarities are more likely to have large compatibilities, we initialize *m*_0_ by normalized first-order tensor matrix *T*_1_. It converges much faster using this strategy.

$$m_{0}\left(i,i^{\prime }\right)=\bar{T_{1}\left(i,i^{\prime }\right)}=T_{1}\left(i,i^{\prime }\right)/\underset{i}{\sum }\underset{i^{\prime }}{\sum }T_{1}\left(i,i^{\prime }\right)$$ (5)

We can derive m* by computing the leading eigenvalue of *T*_*F*_, using Algorithm 1.

**Algorithm 1** Spectral relaxation iterations.

**Input:** minutia tensor matrix ***T***_F_

**Output:** m*, the main eigenvector of ***T***_F_

**1** initialize $m^{*}=m_{0}=\overset{-}{T_{1}}$

**2** repeat

**3** ↓m* ← *T*_*F*_ m*;

**4** $↓m^{*}\leftarrow \frac{1}{{\left||m^{*}|\right|}_{2}}m^{*}$;

**5** until convergence;

Indeed, each step of the iteration algorithm requires only *O* (*z*) operations, where *z* is the number of non-zero elements of the matrix. Also, typically, in our situation, the algorithm converges in a few dozen steps.

Given the continuous solution m*, we can rewrite it in a N_p_ × N_p'_ matrix form and discretize m* by a greedy approach and derive the optimal solution$\tilde{m}$. Because of the existence of partial overlapping, only a subset of size *N*_*S*_ ≤ *min*(*N*_*p*_, *N*_*p'*_) can be matched. Here we set the m* elements whose value is smaller than the pre-set threshold δ to zero. The parameter δ is set manually. Meanwhile, minutia matching is one-to-one matching, minutia *i* in *P* mapped to both minutia *i'* and *j'* in *Q* is impossible. We add this compatibility constraint during the iteration process. It is summarized as Algorithm 2.

**Algorithm 2** Greedy approach for discretization.

**Input:** continuous matrix m*

**Output:** binary matrix $\tilde{m}$

**1** initialize $\tilde{m}=0$

**2** set m* (*i*,*j*) = 0, ∀i, ∀j, m*(*i*,*j*)< δ

**3** repeat

**4** ↓ find the maximal element m*(*i*,*j*)

**5** ↓ set$\tilde{m}(i,j)=1$, ∀k, set m*(*i*,*k*) = 0,m*(*k*,*j*) = 0

**6** until m* = **0**;

The matching constraints (m**1** ≤ **1** and m^T^ **1** ≤ **1**) are ignored in the spectral relaxation step and then are induced during the discretization step. Corrected matched pairs can be gained and similarities of minutia sets can be evaluated. We can get the number *N*_*m*_ of matched pairs.

After that we adjust the similarity score with the number of matched pairs. Take minutia sets *P* and *P'* for example, the similarity *S*_*PP'*_ is calculated using the following formula:

$$S_{PP^{\prime }}=\tilde{M}\cdot N_{m}^{2}/\left(N_{p}\cdot N_{p^{\prime }}\right)$$ (6)

*S*_*ii'*_ measures the similarity of matched pairs *ii'*. $\tilde{M}$indicates the maximum value of the optimization problem shown in formula (3). *N*_*m*_ indicates the number of matched pairs, and *N*_*p*_ and *N*_*p'*_ indicate the minutia number of *P* and *P'*.

In practice, we use the tensor matching strategy both in local matching level and global matching level. There are some differences in these two levels. We will give detailed descriptions in section 2.3 and 2.4.

### 2.3 Local minutia tensor matrix (*Local MTM*)

Local minutia topologic structure (simplified as *LMTS*) is firstly introduced in [12]. A typical *LMTS* is constructed from a center minutia and a list of neighboring minutiae in a specified area. Fingerprint images show rigidity within the range of *LMTS*. Each *LMTS* can be seen as a small minutia set. Here we propose the local *MTM* for minutia structures and design a novel strategy to calculate similarities of *LMTS* pairs.

**Fig 2:**
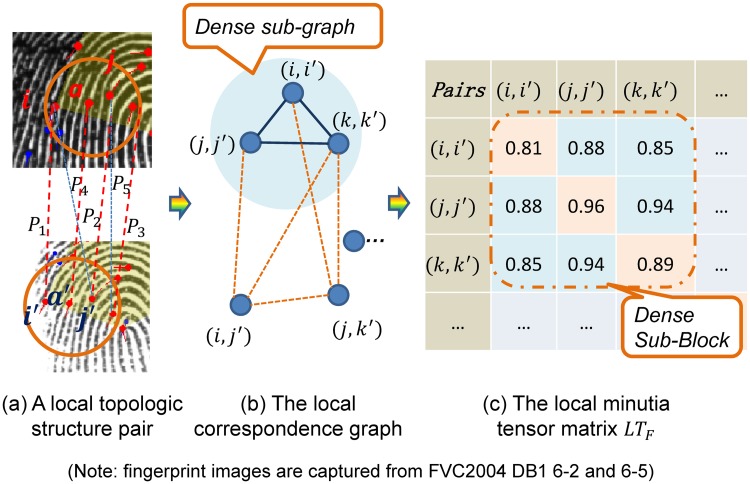
Illustration of local minutia tensor matrix. The fingerprint images are captured from FVC2004 DB1 6–2 and 6–5. Minutia pairs within two local minutia topologic structures shown in (a) form the local correspondence graph in (b) and the local minutia tense matrix (*local MTM*) in (c). Correct pairs corresponds to the dense subgraph of local correspondence graph, and also the dense block of *local MTM*. Minutia matching is formulated as recovering the dense sub-block in the *local MTM*.

As shown in Fig. 2, minutia *a* and *i*, *j*, *k* form a minutia topologic structure *L*_*a*_, minutia *a'* and *i'*, *j'*, *k'* form a minutia topologic structure *L*_*a'*_.

According to the coordinates and orientations of minutiae in *L*_*a*_, we calculate the distance vector $\overset{-}{v}$ between each two minutiae within *L*_*a*_, so it is with *L*_*a'*_. Each candidate minutia pair (*i*, *i'*) is assigned a local similarity attribute LT_1_ (*i*,*i'*) and each two minutia pairs (*ii'*, *jj'*) is assigned a local compatibility attribute LT_2_ (*ii'*, *jj'*). They can be calculated in the following formula:

$$LT_{1}\left(i,i^{\prime }\right)=S_{ii^{\prime }}=\alpha \cdot e^{-\alpha^{\prime }\cdot D\left({\bar{v}}_{ai},{\bar{v}}_{a^{\prime }i^{\prime }}\right)}$$ (7)

$$LT_{2}\left(ii^{\prime },jj^{\prime }\right)=C_{ii^{\prime },jj^{\prime }}=\beta \cdot e^{-\beta^{\prime }\cdot D\left({\bar{v}}_{ij},{\bar{v}}_{i^{\prime }j^{\prime }}\right)}$$ (8)

Where ${\overset{-}{v}}_{ai}$ is the distance vector between minutia *a* and minutia *i*. ${\overset{-}{v}}_{a^{'}i^{'}},{\overset{-}{v}}_{ij},{\overset{-}{v}}_{i^{'}j^{'}}$have similar meanings. Here$D({\overset{-}{v}}_{pi},{\overset{-}{v}}_{p^{'}i^{'}})={\left||{\overset{-}{v}}_{pi}-{\overset{-}{v}}_{p^{'}i^{'}}|\right|}_{1}$. *D* (,) measures the 1-norm distance of vectors ${\overset{-}{v}}_{pi}$ and${\overset{-}{v}}_{p^{'}i^{'}}$. *S*_*ii'*_ indidates the similarity of minutia pair (*i*, *i'*). *C*_*ii'*_,_*jj'*_ indidates the compatibility between pairs (*i*, *i'*) and (j, *j'*). α, α', β, β' are positive parameters. They are set manually.

We calculate all the similarities of minutia pairs in *LT*_1_ and all the compatibilities of each two minutia pairs in *LT*_2_. Thus, we can construct the local first-order tensor matrix *LT*_1_ and the local second-order tensor matrix *LT*_2_. The local fused tensor matrix *LT*_*F*_ (local *MTM*) can be constructed using the method proposed in formula (2). Calculating similarities of *L*_*a*_ and *L'*_*a*_ is formulated as recovering the dense sub-block in the local minutia tensor matrix *LT*_*F*_.

LM~=argmaxLM∑ii'∈LM,jj'∈LMLTF(ii',jj') (9)

In experiments, we make some pruning after building the local *MTM*. Suppose there are two minutia topologic structures *L*_*a*_,*L*_*a'*_, with *n*_*a*_, *n*_*a'*_ minutiae, respectively. Because of the one-to-one mapping constraint, only subsets of size n_s_ ≤min (*n*_*a*_, *n*_*a'*_) can be matched. We retain n_r_ = 3 * min (*n*_*a*_, *n*_*a'*_) candidate minutia pairs with the maximal similarity values. The pruned local *MTM* has only *n*_*r*_ * n_*r*_ elements, which is much smaller than the original scale (*n*_*a*_ * n_*a'*_) * (*n*_*a*_, * n_*a'*_). Then the optimal matching pairs within *LMTS* can be gained through spectral methods proposed in section 2.2.The spectral iteration process is very fast. It usually will converge in several iterations.

After relaxation and discretization, we can gain the optimal matched pairs and the number *n*_*m*_ of matched pairs. After that we adjust the similarity score with the number of matched pairs. The similarity of *LMTS* pairs *L*_*a*_ and *L*_*a'*_ can be calculated using the following formula.

$$S_{L_{a}L_{a^{\prime }}}=\tilde{LM}\cdot n_{m}^{2}/\left(n_{a}\cdot n_{a^{\prime }}\right)$$ (10)

$\tilde{LM}$indicates the maximal score of the local *MTM LT*_*F*_. *n*_*m*_ indicates the number of matched pairs within (*L*_*a*_, *L*_*a'*_), and *n*_*a*_ indicates the number within *LMTS L*_*a*_, *n*_*a'*_ has a similar meaning.

### 2.4 Global minutia tensor matrix (*Global MTM*)

The whole minutia set can be seen as a large global minutia topologic structure and the local minutia topologic structures (*LMTS*s) are basic matching units. Fingerprint images show nonlinearity in the global range. Minutia pairs far apart may not look so compatible. Here we propose the global *MTM* for minutia sets and design a novel strategy to calculate similarities of fingerprints.

**Fig 3:**
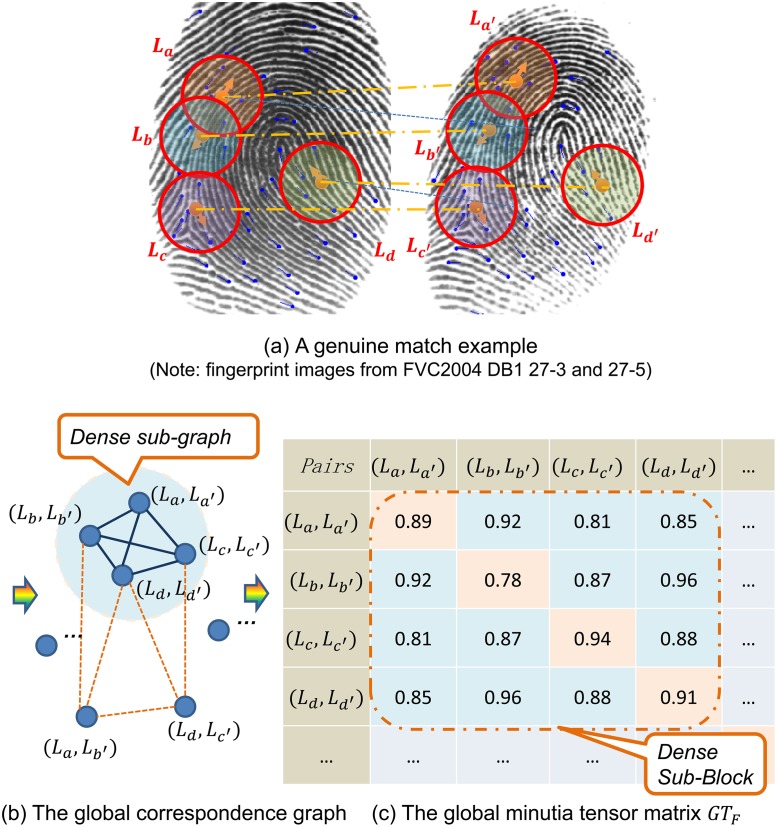
Illustration of global minutia tensor matrix. The fingerprint images are from FVC2004 DB1 27–3 and 27–5. For clarity, only four minutia structure pairs are shown. Candidate structure pairs shown in (a) form the global correspondence graph in (b) and the global minutia tense matrix (*global MTM*) in (c). Correct structure pairs corresponds to the dense subgraph of global correspondence graph, and also the dense block of *global MTM*. Minutia matching is formulated as recovering the dense sub-block in the *global MTM*.

As shown in Fig. 3, L_a_,L_b_,L_c_,L_d_ are four minutia structures in minutia set *P*, L_a'_,L_b'_,L_c'_,L_d'_ are four minutia structures in minutia set *P'*. For clarity, only a small subset of the candidate *LMTS* pairs is shown. *LMTS* pair (*L*_*a*_,*L*_*a'*_) is assigned a global similarity attribute *GT*_1_ (*L*_*a*_, *L*_*a'*_) and each two minutia pairs (*L*_*a*_ *L*_*a'*_,*L*_*b*_ *L*_*b'*_) is assigned a global compatibility attribute *GT*_2_(*L*_*a*_ *L*_*a'*_,*L*_*b*_ *L*_*b'*_).

Fingerprint images show nonlinearity in the global range. Minutia pairs far apart may not look so compatible. We lower the compatibility threshold and take nonlinearity into account. The first-order tensor *GT*_1_(*L*_*a*_,*L*_*a'*_) can be evaluated using the structural similarity $S_{L_{a}L_{a^{'}}}$ gained in section 2.2. According to the coordinates and orientations of *LMTS* center minutiae, we can calculate the distance vector $\overset{-}{v}$ between each two center minutiae, such as${\overset{-}{v}}_{ab},{\overset{-}{v}}_{a^{'}b^{'}}$. The compatibility *GT*_2_(*L*_*a*_ *L*_*a'*_,*L*_*b*_ *L*_*b'*_) can be evaluated using the two pairs of center minutiae (*aa'*, *bb'*). They can be calculated in the following formula:

$$GT_{1}(L_{a},L_{a}^{'})=S_{L_{a}L_{a^{'}}}$$ (11)

$$GT_{2}\left(L_{a}L_{a^{\prime }},L_{b}L_{b^{\prime }}\right)=C_{L_{a}L_{a^{\prime }},L_{b}L_{b^{\prime }}}=C_{aa^{\prime },bb^{\prime }}=\gamma \cdot e^{-\gamma^{\prime }\cdot D\left({\bar{v}}_{ab},{\bar{v}}_{a^{\prime }b^{\prime }}\right)}$$ (12)

Where $S_{L_{a}L_{a^{'}}}$ indidates the similarity of *LMTS* pair (*L*_*a*_, *L*_*a'*_) gained in section 2.3. ${\overset{-}{v}}_{ab}$is the distance vector between center minutiae *a* and *b*. ${\overset{-}{v}}_{a^{'}b^{'}}$has a similar meaning. *C*_*aa'*,*bb'*_ indidates the compatibility between pairs (*a*, *a'*) and (*b*, *b'*). *γ*, *γ'* are positive parameters. They are set manually.

We calculate all the similarities of *LMTS* pairs in *LT*_1_ and all the compatibilities of each two *LMTS* pairs in *LT*_2_. Thus, we can construct the global first-order tensor matrix *GT*_1_ and the local second-order tensor matrix *GT*_2_. The global fused tensor matrix *GT*_F_ (global *MTM*) can be constructed using the method proposed in formula (2). Calculating the similarity of minutia sets *P* and *P'* is formulated as recovering the dense sub-block in the global minutia tensor matrix *GT*_*F*_.

GM~=argmaxGM∑LaLa'∈GM,LbLb'∈GMGTF(LaLa',LbLb') (13)

In experiments, we make some pruning after building the global *MTM*. Suppose there are two minutia sets *P* and *P'*, with *N*_*p*_ and *N*_*p'*_ minutiae, respectively. Because of the one-to-one mapping constraint, only subsets of size *N*_*S*_ ≤ min (*N*_*p*_, *N*_*p'*_) can be matched. We retain *N*_*r*_ = 3 * min (*N*_*p*_,*N*_*p'*_) candidate minutia pairs with the maximal similarity values. The pruned global *MTM* can be gained. It has only *N*_*r*_ * N_*r*_ elements, which is much smaller than the original scale (*N*_*p*_ * N_*p'*_) * (*N*_*p*_ * N_*p'*_). Then the optimal *LMTS* pairs can be gained through spectral methods proposed in section 2.2.The spectral iteration process is very fast. It usually will converge in several iterations.

After relaxation and discretization, we can gain the optimal matched pairs and the number *N*_*m*_ of matched pairs. After that we adjust the similarity score with matched pairs. As isolated minutiae or those minutiae in the border of fingerprint images have few surrounding minutiae, they may be removed at the step of constructing *LMTS*s. It is necessary to regain the lost matches. We use the moving least squares (*MLS*) model proposed in [26] to regain lost genuine minutia pairs. First, the parameters of *MLS* deformation are estimated from the matched minutia pairs. Then the estimated parameters are used to transform the model minutia set to the target minutia set. Third, a tolerance box is adopted to scan each minutia. When two minutiae from different fingerprints drop into the same tolerance box, they are judged as matched. For fingerprints from the same finger, many genuine minutia pairs can be retrieved. For fingerprints from different fingers, few minutiae can be regained as the randomness of minutiae’s positions. In this way we can get the final matched pairs.

Here we design a new scoring strategy to get the final matching scores. We adopt the convex hull strategy proposed in [17] to adjust the final similarity score. The convex hull can be gained and the number of unmatched points within the convex hull can be calculated. The evaluation strategy is based on convex hull and can be represented as follows:

$$S_{PP^{\prime }}=\tilde{GM}\cdot N_{m}^{2}/\left(\left(N_{m}+m_{p}\right)\cdot \left(N_{m}+m_{p^{\prime }}\right)\right)$$ (14)

$\tilde{GM}$indicates the maximal score of the global *MTM GT*_*F*_. *N*_*m*_ indicates the number of matched points, and *m*_*p*_ indicates the number of unmatched points within the convex hull in minutia set *P* and *m*_*p'*_ indicates the number of unmatched points within the convex hull in minutia set *P'*. It is more reasonable to adopt this new score evaluation strategy.

## Algorithm

In this section, we describe our proposed algorithm in detail. It mainly contains the following parts: initialization, local matching and global matching.

### Initialization

Suppose there are two minutia sets *P* and *P'*, with *N*_*p*_ and *N*_*p'*_ minutiae, respectively. For each minutia *i* in *P*, we construct the *LMTS* with *i* as the center minutia and a list of neighboring minutiae in the circular area. In this way, *N*_*p*_ *LMTS*s in *P* can be constructed. We take the same operations in *P'* and *N*_*p'*_ *LMTS*s can be constructed, too. According to the coordinates and orientations of minutiae, we calculate all the distance vectors ${\overset{-}{v}}_{ij},(\forall i\in P,j\in P)$ within minutia *P* and all the distance vectors ${\overset{-}{v}}_{i^{'}j^{'}},(\forall i^{'}\in P^{'},j^{'}\in P^{'})$ within minutia *P'*. We calculate all the similarities of minutia pairs (*i*, *i'*), ∀*i* ∊ *P*, ∀*i*' ∊ *P'* using the formula (7). Meanwhile, we calculate all the compatibilities of each two pairs (*ii'*, *jj'*), ∀*i*,*j* ∊ *P*, ∀*i*', *j'* ∊ *P'* using the formula (8). In this way, we can gain *N*_*p*_ *× N*_*p'*_ similarities and (*N*_*p*_ *× N*_*p'*_) × (*N*_*p*_ *× N*_*p'*_) compatibilities in all.

### Local matching

For minutia sets *P* and *P'*, with *N*_*p*_ and *N*_*p'*_ minutiae, we construct *N*_*p*_ and *N*_*p'*_ *LMTS*s, respectively, thus we gain *N*_*p*_ × *N*_*p'*_ *LMTS* pairs. For each *LMTS* pair from 1 to *N*_*p*_ × *N*_*p'*_, we conduct a local matching operation.

The local matching operation contains three steps:

(a) Building the local *MTM*: For the *LMTS* pair (*L*_*a*_, *L*_*a'*_), we select the corresponding minutia pairs within *L*_*a*_ and *L*_*a'*_. (The similarities and compatibilities have been calculated in the initialization step and we just need to select them.) We build the local first-order tensor matrix *LT*_1_ and the local second-order tensor matrix *LT*_2_. Next we construct the local *MTM LT*_F_ using formula (2).

(b) Spectral matching: Calculating the similarity of *LMTS* pair (*L*_*a*_, *L*_*a'*_) yields to the optimization problem shown in formula (9). We use the spectral matching methods proposed in section 2.2 to deal with it. The maximal value $\tilde{LM}$ and optimal matching pairs can be gained.

(c) Scoring: After gaining the maximal $\tilde{LM}$ and the optimal matching pairs, we calculate the similarity $S_{L_{a}L_{a^{'}}}$ of *LMTS* pair (*L*_*a*_, *L*_*a'*_) using formula (10).

After *N*_*p*_ *× N*_*p'*_ local matching operations, we can gain all the similarities of candidate *LMTS* pairs.

### Global matching

(a) Building the global *MTM*: After gaining the similarities of all candidate *LMTS* pairs, we can build the global first-order tensor matrix *GT*_1_. For each two *LMTS* pairs (*L*_*a*_ *L*_*a'*_, *L*_*b*_ *L*_*b'*_), we evaluate their compatibility through their center minutiae (*aa'*, *bb'*). The calculation function can be seen in formula (12). In this way, (N_p_ × N_p'_) × (N_p_ × N_p'_) *LMTS* compatibilities can be calculated and the global second-order tensor matrix *GT*_2_ can be built. Next we construct the global *MTM GT*_F_ with (N_p_ × N_p'_) × (N_p_ × N_p'_) elements using formula (2).

(b) Spectral matching: Calculating the similarity of minutia sets *P* and *P'* yields to the optimization problem shown in formula (13). We use the spectral matching methods proposed in section 2.2 to deal with it. The maximal value $\tilde{GM}$ and the optimal *LMTS* pairs can be gained.

(c) Regaining: After calculating the maximal value $\tilde{GM}$ and the optimal *LMTS* pairs, we regain lost genuine pairs using the *MLS* strategy. In this way the final matched pairs can be gained.

(d) Scoring: After gaining the maximal value $\tilde{GM}$ and the final matched pairs. We adopt the convex hull strategy to adjust the final similarity score. We calculate the entire similarity *S*_*PP'*_ of minutia sets *P* and *P'* using formula (14).

The novel fingerprint matching algorithm is summarized as algorithm 3.

**Algorithm 3** High-order tensors matching algorithm.

**Input:** Two fingerprint minutia sets *P* and *P'*, with ***N***_p_ and ***N***_p'_ minutiae, respectively.

**Output:** The entire similarity ***S***_PP'_ of minutia sets ***P*** and ***P'***

**1** Initialization:

  constructing local minutia topologic structures:

***N***_p_ and ***N***_p_'structures, respectively.

  calculating similarities and compatibilities of all minutia pairs:

***N***_p_ ***× N***_p'_ similarities and **(*N***_p_ ***× N***_p'_ **)×(*N***_p_ ***× N***_p'_) compatibilities.

**2** Local matching: calculating *LMTS* similarities

 repeat: from 1 to ***N***_p_ ***× N***_p'_

  calculating similarity of *LMTS* pairs for *LMTS* pairs (*L*_*a*_,*L*_*a'*_)

  building the local first-order tensor ***LT***_1_ and the local second-order tensor ***LT***_2_

  constructing the local minutia tensor matrix ***LT***_*F*_

  spectral matching for $\tilde{LM}$ and matched pairs

  calculating the similarity $S_{L_{a}L_{a^{'}}}$ of *LMTS* pair (*L*_*a*_,*L*_*a'*_)

 until all candidate *LMTS* pairs are calculated.

**3** Global matching: calculating entire similarity

 building the global first-order tensor ***GT***_1_and the global second-order tensor ***GT***_2_

 constructing the global minutia tensor matrix ***GT***_*F*_

 spectral matching for $\tilde{GM}$ and matched pairs

 regaining lost genuine minutia pairs using *MLS* model

 constructing the convex hulls of minutia sets ***P*** and ***P'***.

 calculating the entire similarity ***S***_***PP'***_ of minutia sets ***P*** and ***P'***

## Results

### 4.1 Fingerprint Database

Comparative experiments are conducted on the International Fingerprint Verification Competition (simplified as *FVC*) databases: *FVC2002* and *FVC2004* databases. *FVC2002* contains four different sub-databases and each database has 800 fingerprints (100 fingers and 8 impressions per finger) [2]. For each sub-database, each two impressions per finger are tested and there are 2800 true matches. The first impressions of each two fingers are tested and there are 4950 fake matches. We conduct 7750 matches in all. It is the same with *FVC2004* [3]. They are international competition databases for fingerprint verification algorithms, which are open to all researchers (*FVC2002* can be downloaded in http://bias.csr.unibo.it/fvc2002/ and *FVC2004* can be downloaded in http://bias.csr.unibo.it/fvc2004/). The image quality of *FVC2002* is good, while the image quality of FVC2004 is relatively bad. It contains much noise and nonlinearity.

For each algorithm and for each dataset, the following performance indicators are considered: Equal-Error-Rate (simplified as *EER*); False Match Rate (simplified as *FMR*); False Non Match Rate (simplified as *FNMR*); the lowest *FNMR* for *FMR* ≤ 0.1% (simplified as *FMR-1000*), average time for per match (simplified as *Time*) and the corresponding receiver operating characteristic curves *(*simplified as *ROC cures*). These experiments run on Intel i5–760 (2.80 GHz) processor (single-thread) and 32-bit Windows 7 systems.

### 4.2 Experiments

**Fig 4:**
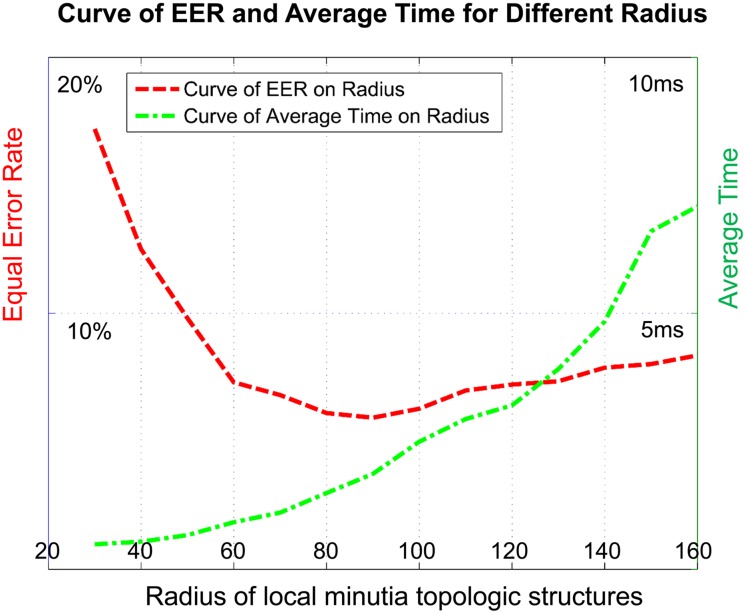
Curves of *EER* and *Time* with the growth of structural radius on *FVC2004-DB1*.

The matrix scale of *local MTM* plays an important role for local matching. It is directly affected by the radius of *LMTS*. Here we make a special experiment on *FVC2004*-*DB1* to test the influence. We use the proposed matching algorithm and vary the radius size from 30 pixels to 160 pixels. We observe the relationship between *EER*, average time and the *LMTS* radius. Fig. 4 shows the results. The radius ranges from 30 pixels to 160 pixels. The average time increases with the radius length as the matrix scale of *MTM* is directly affected by the radius of *LMTS*. *EER* decreases at first and then increases with the radius. It is because that fingerprints show rigidity in local areas and nonlinearity in global areas.

In the following experiments, we set each parameter the same value for all algorithms. The *LMTS* radius is set to 80 pixels. The matrix fusion parameter *λ* in formula (2) is set to 0.1. The minimal similarity threshold *δ* in section 2.2 is set to *e* ^-4^. The local similarity parameters α, α' in formula (7) is set to 1, 0.5, respectively. The local compatibility parameters *β*, *β'* in formula (8) is set to 2, 0.33, respectively. The global compatibility parameters *γ*, *γ'* in formula (12) is set to 2, 0.2, respectively.

Comparative experiments are conducted on *FVC2002* and *FVC2004* databases. In order to demonstrate the effectiveness of the new algorithm, some other matching algorithms are used, including the efficient minutia-based fingerprint matching algorithm (simplified as *EMF*) [8], the local structural similarity algorithm (simplified as *LSS*) [11], the local topologic structure algorithm (simplified as *LTS*) [12], the grow and fuse algorithm (simplified as *GF*) [14], the minutia cylinder-code algorithm (simplified as *MCC*) [15], the minutiae triplets algorithm (simplified as *MT*) [16] and the local structure compatibility algorithm (simplified as *LSC*) [18]. Here we use only minutia features (coordinates and orientations) for every algorithm. We do not use other matching features such as qualities or ridges to ensure the comparison effectiveness.

The proposed algorithm has two levels: local *MTM* matching and global *MTM* matching. We make validation for each level, respectively.

#### 4.2.1 Validation of *local MTM*

*Local MTM* is proposed to calculate the similarity of *LMTS*s. In order to test the effectiveness of the local matching part, we do a special group of experiments on *FVC* databases. Four different methods for measuring *LMTS* are used: the local matching part of *LSS* (simplified as *local LSS*) [11], the local matching part of *LTS* (simplified as *local LTS*) [12], the local matching part of *MCC* (simplified as *local MCC*) [15] and the proposed local *MTM*. We choose the four algorithms for local matching as all of them have the step of local matching. *Local LSS* used a matching strategy based on graph representation. *Local LTS* used a longest common subsequence strategy to calculate the similarity of two *LMTS*s. *Local MCC* proposed a novel representation based on 3D data structures. For *local MTM*, we only use the local matching part proposed in section 2.3.

In order to ensure the comparison effectiveness of these four methods, we use the four different methods to measure similarities of *LMTS* pairs, and then using the same post-processing operations. After gaining the similarity matrix of *LMTS* pairs, we discard the different post-processing operations of these original algorithms and use the same discretization operation proposed in algorithm 2 to gain the optimal matching pairs. The final scores are simply evaluated using the same convex hull strategy, which is shown in the following formula.

$$S_{PP^{'}}=\sum_{matched}S_{LL^{'}}\cdot \frac{N_{m}^{2}}{\left(N_{m}+m_{p})\cdot (N_{m}+m_{p^{'}}\right)}$$ (15)

In this formula *S*_*LL'*_ indicates the similarity of *LMTS* pair (*L*, *L'*), *N*_*m*_ indicates the number of matched pairs. *m*_*p*_ indicates the number of unmatched points within the convex hull in minutia set *P* and *m*_*p'*_ indicates the number of unmatched points within the convex hull in minutia set *P'*.

For each algorithm and for each dataset, the performance indicator of *EER* and *Time* is considered. *EER* Results on *FVC2002* and *FVC2004* are shown in the Table 1. The experimental results show that our *local MTM* algorithm is close to well-known algorithm *MCC* in the *EER* indicator. It has promising performances in terms of both the *EER* and *FMR-1000*. Meanwhile, *Time* results on *FVC2004-DB1* is shown in Table 2. We can see that the proposed *local MTM* is better than other algorithms in the *Time* indicator. Specially, the proposed *local MTM* is much better than *local MCC*, as we don’t need to construct minutia structures as complex as *local MCC*.

**Table 1:** *EER* results on FVC database for local matching.

	D1	D2	D3	D4	D*1	D*2	D*3	D*4
*local LSS*	3.54	4.28	5.21	3.21	9.38	12.56	9.54	7.91
*local LTS*	2.36	2.68	3.34	2.34	8.16	10.81	8.34	6.21
*local MCC*	1.47	**1.82**	**1.89**	1.65	6.25	**8.54**	6.32	5.32
*local MTM*	**1.46**	2.25	2.47	**1.34**	**5.36**	8.97	**6.29**	**5.15**

*Local LSS*, *local LTS*, *local MCC* and *local MTM* are four algorithms for local matching. D1, D2, D3 and D4 are four sub-databases of *FVC2002*. D*1, D*2, D*3 and D*4 are four sub-databases of *FVC2004*.

**Table 2:** *Time* on *FVC2004-DB1* for local matching.

	*local LSS*	*local LTS*	*local MCC*	*local MTM*
*Time*	0.62	0.88	2.41	**0.57**

*Local LSS*, *local LTS*, *local MCC* and *local MTM* are four algorithms for local matching. This table shows the average matching time (*ms*) for per match on *FVC2004-DB1*.

#### 4.2.2 Validation of *global MTM*

From the experiments for local matching we can see, *MCC* and the proposed local *MTM* are two better methods for measuring similarities of *LMTSs* pairs. They can be used to calculate the structural similarity for global matching. Take *global MTM* into consideration, we design two combined algorithms: *local MCC* + *global MTM*, and *local MTM* + *global MTM*. We compare these two algorithms with some other algorithms, including *EMF* [8], *LSS* [11], *LTS* [12], *GF* [14], *MCC* [15], *MT* [16] and *LSC* [18]. *EMF* applied a set of global level minutia dependent features including qualities and the area of overlapping regions. Here we use only the area of overlapping regions for fairness. *LSS* used a matching technique based on graph representation and then an expanding strategy based on distances. *LTS* proposed a novel matching algorithm based on local topologic structures and then used a novel method to compute the similarity. *GF* proposed a robust matching approach based on the growing and fusing of local structures. *MCC* proposed a novel representation based on 3D data structures. *MT* proposed a novel fingerprint matching algorithm named *M3gl*. *LSC* proposed the concept of compatibility to the minutiae triangle structures and adopted a relaxation process for global adjustment. In order to ensure the comparison effectiveness of these four methods, the radius of minutia structures is set to 80 pixels equally.

**Fig 5:**
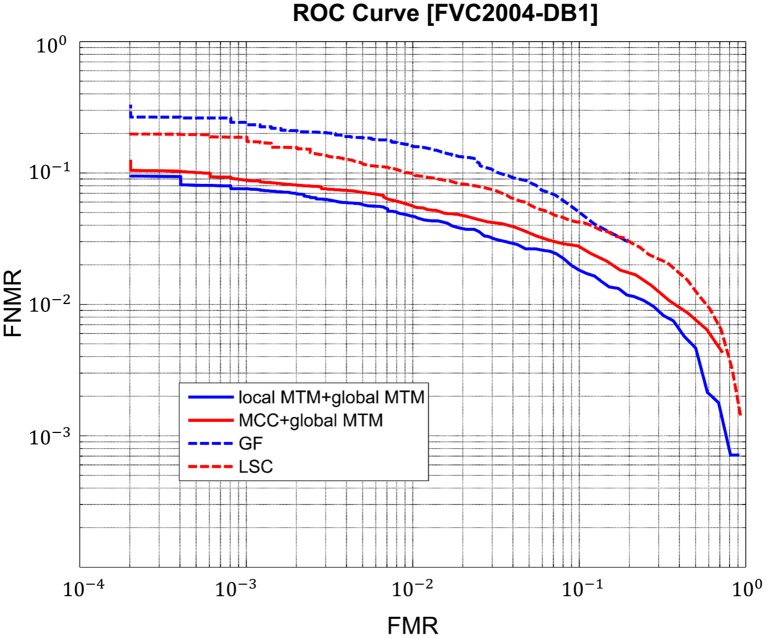
*ROC* curves on *FVC2004-DB1* for global matching.

For each algorithm and for each dataset, the performance indicators of *EER*, *FMR-1000*, *Time* are considered. Results on *FVC2002* and *FVC2004* are shown in the Table 3 and Table 4. The corresponding *ROC curves* for *FVC2004-DB1* are shown in Fig. 5.

**Table 3:** Comparative results on *FVC2002* database for global matching.

	D1-E	D1-F	D2-E	D2-F	D3-E	D3-F	D4-E	D4-F
*EMF*	0.94	2.12	0.86	2.37	1.35	4.78	0.78	2.88
*LSS*	2.07	4.98	2.89	6.85	3.91	9.28	1.87	4.76
*LTS*	1.85	3.23	2.33	5.21	3.12	8.22	1.63	3.77
*MT*	1.10	2.30	1.30	1.90	3.10	7.50	2.40	5.60
*MCC*	0.58	1.02	**0.67**	1.93	1.17	3.53	0.31	2.24
*LSC*	2.54	5.14	3.76	8.46	3.15	5.27	1.98	4.54
*GF*	1.08	1.27	1.32	1.94	2.15	5.31	0.62	2.31
*MCC*+*MTM*	**0.42**	**0.69**	0.85	1.95	1.27	**3.23**	0.21	0.94
*MTM*+*MTM*	0.57	0.98	**0.71**	**1.92**	**1.08**	3.87	**0.19**	**1.89**

*EMF*, *LSS*, *LTS*, *MT*, *MCC*, *LSC*, *GF*, *MCC* + *MTM*, *MTM* + *MTM* are nine algorithms for global matching. D1, D2, D3 and D4 are four sub-databases of *FVC2002*. D1-E indicates the *EER* indicator for *FVC2002* DB1. D1-F indicates the *FMR-1000* indicator for *FVC2002* DB1.

**Table 4:** Comparative results on *FVC2004* database for global matching.

	D*1-E	D*1-F	D*2-E	D*2-F	D*3-E	D*3-F	D*4-E	D*4-F
*EMF*	5.33	15.83	6.72	15.02	4.23	8.85	3.29	7.69
*LSS*	6.23	17.01	6.81	17.21	6.09	15.21	5.32	11.87
*LTS*	5.88	16.32	5.99	11.72	4.82	8.92	3.38	8.26
*MT*	6.30	19.30	6.20	13.60	6.10	14.40	3.00	6.90
*MCC*	4.30	9.84	5.37	9.26	2.55	6.08	1.69	5.98
*LSC*	9.02	17.69	10.31	25.24	5.25	9.23	6.87	12.37
*GF*	4.21	8.824	4.91	9.21	3.22	8.38	3.91	6.34
*MCC*+*MTM*	3.77	9.12	5.94	11.13	2.25	5.65	**1.59**	**4.11**
*MTM*+*MTM*	**3.13**	**7.54**	**3.62**	**7.914**	**2.17**	**5.39**	1.62	5.48

*EMF*, *LSS*, *LTS*, *MT*, *MCC*, *LSC*, *GF*, *MCC* + *MTM*, *MTM* + *MTM* are nine algorithms for global matching. D*1, D*2, D*3and D*4 are four sub-databases of *FVC2004*. D*1-E indicates the *EER* indicator for *FVC2004* DB1. D*1-F indicates the *FMR-1000* indicator for *FVC2004* DB1.

**Fig 6:**
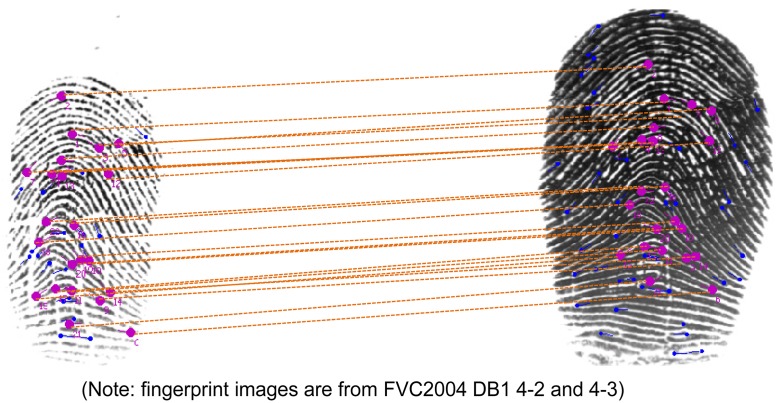
A true match from *FVC2004-DB1* 4–2 and 4–3.

We can get two conclusions: First, the combined two algorithms based on *global MTM* outperforms the other algorithms. Second, the algorithm based on *local MTM* and *global MTM* has the best results. It has promising performances in terms of both the *EER*, *FMR-1000* and *Time*. Fig. 6 shows a true match with much noise and large nonlinearity. Thanks to the strong robustness of new method, many true matches are still found and they are judged as matched.

For *FVC2004-DB1*, there are 800 fingerprints. Each two impressions per finger are tested and there are 2800 true matches. The first impressions of each two fingers are tested and there are 4950 fake matches. The average matching time for *FVC2004-DB1* is shown in Table 5. The result shows that our algorithm is significantly efficient. It is reasonable as both *local MTM* and *global MTM* run very fast. We don’t need to construct minutia structures as complex as *MCC*.

**Table 5:** Time on *FVC2004-DB1* for global matching.

	*EMF*	*LSS*	*LTS*	*MT*	*MCC*	*LSC*	*GF*	MCC+MTM	*MTM*+*MTM*
*Time*	1.87	1.09	1.23	3.26	4.12	1.63	2.37	3.46	**1.02**

*EMF*, *LSS*, *LTS*, *MT*, *MCC*, *LSC*, *GF*, *MCC* + *MTM*, *MTM* + *MTM* are nine algorithms for global matching. This table shows the average matching time (*ms*) for per match on *FVC2004-DB1*.

#### 4.2.3 Comparative Experiments with our previous papers

As we have described in the introduction part, this paper is innovative on the basis of two previous papers [24] [25]. We firstly applied the spectral matching strategy (simplified as *SM*) to global matching [24]. The spectral method was only applied to global matching, while other strategies were still required in local matching. Afterwards, we proposed the extended clique models (simplified as *ECM*) to deal with local matching and global matching [25].The process in local matching is time-consuming. Meanwhile, local matching is not associated with global matching. This paper firstly unifies local matching and global matching into an integrated framework. We design local *MTM* for local matching and global *MTM* for global matching, respectively. It is efficient in both levels. We conduct a group of experiments for the previous *SM*, *ECM* algorithms and the proposed *MTM* algorithm.

Results on *FVC2002* and *FVC2004* are shown in Table 6 and Table 7. The experimental results show that our *local MTM* algorithm has the best performances in terms of both the *EER* and *Time*. The *ECM* algorithm has promising effectiveness, but low efficiency.

**Table 6:** Comparative results between MTM and previous papers.

	D1	D2	D3	D4	D*1	D*2	D*3	D*4
*SM*	1.02	0.98	1.85	0.57	5.97	6.98	3.67	4.39
*ECM*	0.68	**0.67**	1.28	0.20	3.33	3.98	2.88	2.21
*MTM*	**0.57**	0.71	**1.08**	**0.19**	**3.13**	**3.62**	**2.17**	**1.62**

*SM*, *ECM* and *MTM* are three algorithms for fingerprint matching. D1, D2, D3 and D4 are four sub-databases of *FVC2002*. D*1, D*2, D*3and D*4 are four sub-databases of *FVC2004*.

**Table 7:** Time between MTM and previous papers.

	*SM*	*ECM*	*MTM*
*Time*	2.75	1.93	**1.02**

*SM*, *ECM* and *MTM* are three algorithms for fingerprint matching. This table shows the average matching time (*ms*) for per match on *FVC2004-DB1*.

## Discussion

### 5.1 Effectiveness of this method

The proposed algorithm, including *local MTM* and *global MTM*, has promising effectiveness. It fully takes fingerprint characteristic into consideration. The *local MTM* is proposed to describe local rigidity. It contains similarities and compatibilities of minutia pairs within *LMTS*s. The *global MTM* is proposed to deal with global nonlinearity. It contains similarities and compatibilities of *LMTS* pairs within two minutia sets. For true minutia pairs, most of them have both high similarities and high compatibilities, thus they will be judged as matched. Some of them may have high similarities and low compatibilities due to the nonlinearity, they will still be selected in the *global MTM* and judged as matched. Some of them may have low similarities and high compatibilities due to the sparseness or noise, they will be excluded at the local matching step firstly and then regained at the global matching step. True minutia pairs with both low similarities and low compatibilities are unsolvable. They may be regained through other matching features, such as ridges.

### 5.2 Efficiency of this algorithm

The proposed algorithm has promising efficiency. Minutia matching is formulated as seeking for the dense sub-block in the corresponding tensor matrix. Fingerprint matching is divided into two levels: local matching and global matching. In each level it contains two steps: building the tensor matrix and spectral iteration. We make some pruning when building the tensor matrixes. The pruned tensor matrix is much smaller than original matrix. The time for building tensor matrixes is acceptable. Meanwhile, the spectral relaxation is very fast, it usually converges in 20 iterations. Suppose there are two minutia sets *P* and *P'*, with *N*_*p*_ and *N*_*p'*_ minutiae, respectively, this algorithms contains these operations: *N*_*p*_ × *N*_*p'*_ times of local spectral optimization at the local matching step, one time of global spectral optimization at the global matching step, a *MLS* expanding operation and a convex hull operation. Each operation runs very fast.

### 5.3 Future work

As we have described in section 4.1, true minutia pairs with both low similarities and low compatibilities are unsolvable. They may be regained through other matching features, such as qualities and ridges. Our future work shall focus on: (1) adding new matching features, such as phase difference feature, ridges; (2) taking minutia quality into consideration. We will try to add other features into our algorithm framework.

## Conclusions

In this paper, we propose the novel tensor strategy for fingerprint matching. First, the concept of minutia tensor matrix is proposed. It unifies both the first-order features and the second-order features appropriately. Second, correct minutia pairs correspond to the dense sub-block in the *MTM*. Minutia matching is formulated as recovering the main dense sub-block. Spectral matching methods are then used for recovering the dense sub-block. It is efficient and effective. Third, two different kinds of *MTM*s are constructed. The local *MTM* is constructed to calculate similarities of *LMTS* pairs. It makes use of local rigidity. The global *MTM* is constructed to calculate entire similarity. It takes global nonlinearity into consideration.

In summary, this paper proposes a novel structural similarity calculation and a novel approach for fingerprint recognition. It makes better use of similarities and compatibilities. It has stronger description ability and better robustness to nonlinearity and noise. Experimental results demonstrate the effectively and accuracy of our algorithm.
